# Deep learning-based inverse design of microstructured materials for optical optimization and thermal radiation control

**DOI:** 10.1038/s41598-023-34332-3

**Published:** 2023-05-06

**Authors:** Jonathan Sullivan, Arman Mirhashemi, Jaeho Lee

**Affiliations:** 1 Department of Mechanical and Aerospace Engineering, University of California, Irvine, CA USA; 2 NASA Glenn Research Center, Cleveland, OH USA

**Keywords:** Nanophotonics and plasmonics, Metamaterials, Mechanical engineering

## Abstract

Microstructures with engineered properties are critical to thermal management in aerospace and space applications. Due to the overwhelming number of microstructure design variables, traditional approaches to material optimization can have time-consuming processes and limited use cases. Here, we combine a surrogate optical neural network with an inverse neural network and dynamic post-processing to form an aggregated neural network inverse design process. Our surrogate network emulates finite-difference time-domain simulations (FDTD) by developing a relationship between the microstructure’s geometry, wavelength, discrete material properties, and the output optical properties. The surrogate optical solver works in tandem with an inverse neural network to predict a microstructure’s design properties that will match an input optical spectrum. As opposed to conventional approaches that are constrained by material selection, our network can identify new material properties that best optimize the input spectrum and match the output to an existing material. The output is evaluated using critical design constraints, simulated in FDTD, and used to retrain the surrogate—forming a self-learning loop. The presented framework is applicable to the inverse design of various optical microstructures, and the deep learning-derived approach will allow complex and user-constrained optimization for thermal radiation control in future aerospace and space systems.

## Introduction

Engineering surfaces at the microscopic level enable control over the material’s matter-light interactions, and is integral to evolving technologies in areas such as sub-ambient passive cooling^1–4^, radiative heating^5,6^, and thermophotovoltaics^7,8^. The design of selective emitters for radiative thermal management systems depends on two photonic wavelength (λ) spectrums: the visible (VIS) to near-infrared (NIR) and the mid-infrared (MIR)^1,4^. Passive cooling structures—materials that can passively cool beneath ambient temperatures—are surfaces engineered from materials such as polymers^2,3,9^, composites^10–12^, and graphene^13,14^ to maximize thermal emission in the MIR and minimize absorbed solar radiation (λ = 300–2500 nm) by increasing reflected solar radiation. Methods such as nanostructuring^15^, corrugated structures^6,16^, core–shell materials^17,18^, and periodic gratings^16,19^ can be used to induce the opposite effect and increase thermal absorption by enhancing a surface’s anti-reflective behavior. A method that can be used to engineer both radiative heating and cooling materials is microscale “micropyramid” surface texturing^20^. A form of surface relief grating, micropyramid texturing induces anti-reflective properties due to significant light confinement via the combination of material and geometry^20–22^. This method can significantly enhance broadband anti-reflective properties in silicon^23–27^, metals^5,28–32^, dielectrics^33^, and polymers^34^.

Designing and optimizing structures to selectively control optical properties can be a significant and time-consuming challenge. Beyond the potential for many degrees of freedom in the geometric design space, material selection adds an additional level of complexity. Solving the interplay between a complex geometry and material selection can require both a significant investment in computational resources and a dedicated numerical method such as a finite-difference time-domain (FDTD)^35^ solver. A highly effective method that has emerged to counteract the necessity of complex simulation tools is the use of Deep Learning (DL) to predict optical properties. A branch of machine learning (ML), DL methods have shown to have a high degree of non-linear abstraction from datasets^36^ and to address complex issues such as self-driving cars^37^, speech recognition^38^, and natural language processing^39^. Deep Learning has been used in the field of photonics and nanophotonics to predict and model problems such as plasmonic interactions^36,40^, grating structures^41–43^, particles^44,45^, and nanostructures^46^. DL has also been extensively applied within the field of thermal engineering to study topics such as thermal conductivity^47^, boiling heat transfer^48^, and radiative thermal transport^49–51^.

Deep Learning has proven to be not only effective in predicting the “forward” problem by replacing the optical solution process, but also in performing inverse design^36,44,45,52–56^. Inverse design, broadly, is taking a desired input and outputting a set of features that generate the input. Compared to common optimization tools, inverse design via machine learning methods is highly effective in increasing throughput and prediction speed. A multitude of methods exist for executing an inverse design scheme in nanophotonics^57^, but several common methods include the use of a surrogate model in conjunction with an optimization method^58,59^, the creation of a the “tandem” or bidirectional scheme^36,44,55,60,61^, and adversarial networks^62,63^. Inverse design methods based on machine learning have been applied to the design of selective emitter structures; methods based on images^64^, deep learning^65^, deep-binary search^66^, transfer learning^67^, and genetic algorithms^68,69^ have been shown to be effective in previous studies. While some studies do factor in the material selection as an output in the inverse design process, they are often limited to a small set of fixed material outputs^44,67^.

Material selection plays a fundamental role in the design of selective emitters as the interaction of light with the surface is regulated by the spectral material properties^70^. If a microstructure or material is not capable of regulating certain wavelengths, a designer can coat additional material(s) to enhance the broadband response^17,31,32,71^, create a new composite material^11,12^, or select a new material as the basis for patterning. Thus, it is critical to be able to exhaustively search over the available material space to provide the best match for a given set of thermal design criteria^72^. To be exhaustive in the inverse design approach, the material output cannot be fixed and should be flexible to allow the discovery of unique combinations of material properties with geometric properties.

In this work, we propose an inverse microstructural design method based on a tandem neural network constructed to take in a set of desired optical properties and output a set of material and geometric properties. We supplement the tandem neural network—consisting of a surrogate network and inverse network—with post-processing methods to allow the aggregated network to consider critical physical design constraints. The aggregated network is designed in an adversarial style process loop to facilitate the model to iterate and build upon itself over subsequent generations and consider focused feedback from the post-processing checkpoints. The foundation of our method is built upon a previously developed optical simulation surrogate based on a deep neural network (DNN)^72^. As opposed to many other studies that provide a deep learning approach to optics where a single material is simulated^73^, the materials are fixed^41^, or are one-hot encoded^44^, our surrogate method does not constrain material input and can extrapolate to make predictions for materials that were not used in training. The flexibility provided by this method enables us to build an inverse neural network structure to work in tandem with the surrogate that is similarly unconstrained by material classification. Our inverse network structure is capable of not only predicting an optimal material for a given desired input but is capable of extrapolating new material properties to match a given desired input.

The model we demonstrate takes in a simple input of optical properties across a wavelength range and outputs a material and micropyramid geometry that best match it. Multiple deep learning methods are utilized in the construction of the method. We compare the previously established deep neural network surrogate to an image-based surrogate and incorporate recurrent neural network functionality to improve the inverse network’s prediction performance. While we demonstrated limited optimization using the surrogate^72^, the inverse network enables far more rapid, dynamic, and global optimization. The output of the inverse neural network is put through a post-processing stage where user set geometric and material constraints are used to produce appropriate solutions. The novel generated material properties are matched to a material from a library material, the constrained output is simulated, and the results are incorporated in the surrogate network. Using this process, we can rapidly optimize a material and geometric combination for a desired optical spectrum in a process that would be too computationally expensive to perform otherwise. While we apply our methodology to micropyramid structures, the approach that we demonstrate can be modified to accommodate any number of microstructural surface topologies.

## Results

**Figure 1:**
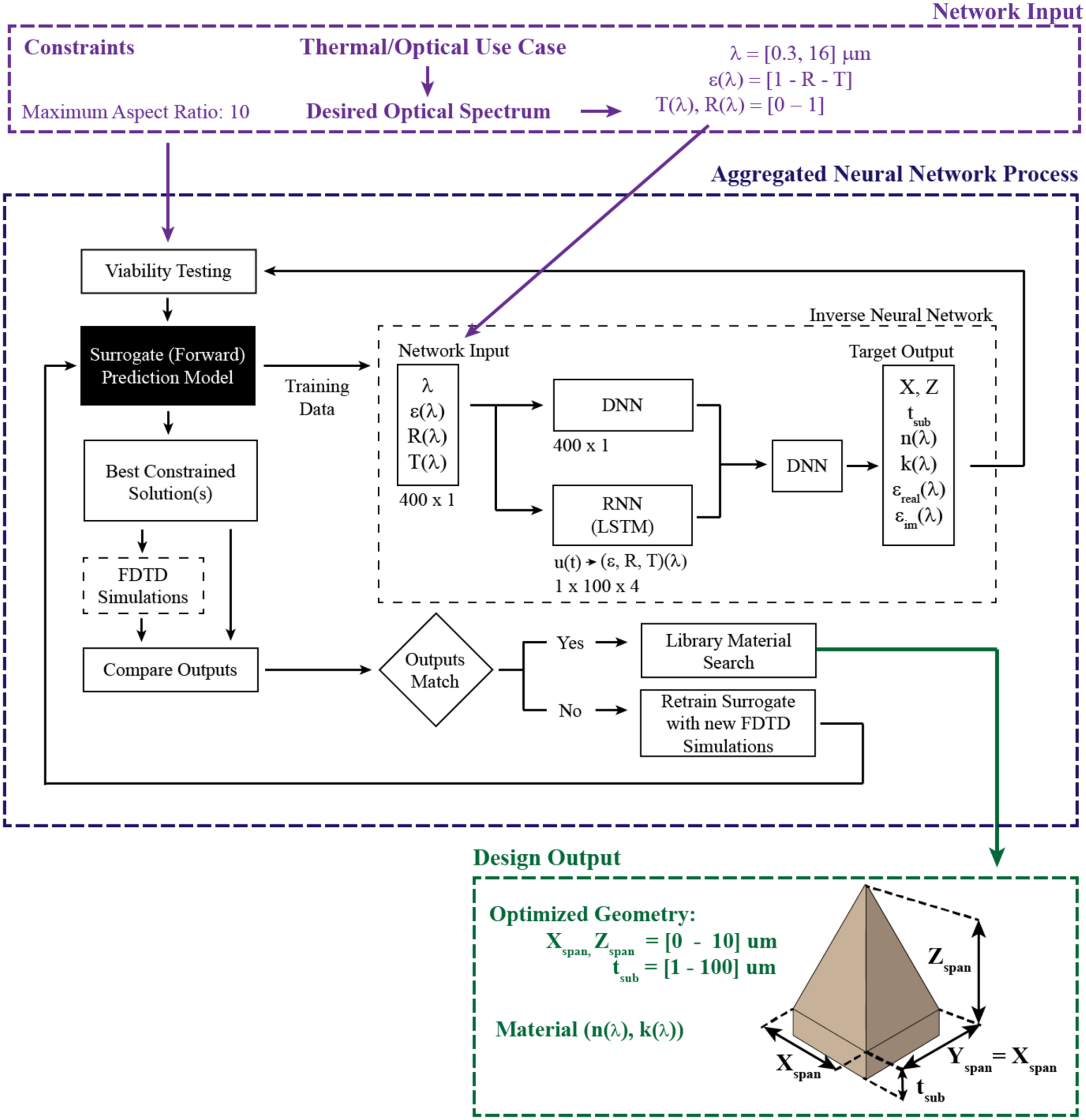
Flow-chart representation of aggregated neural network methodology and inverse network architecture. Solutions generated in the surrogate (forward) solution are used to train the inverse solver. The input to the inverse neural network is a 400 × 1 vector of wavelength, and the wavelength dependent emissivity, reflectivity, and transmissivity. The output of the inverse neural network is a set of material properties that correspond to the input wavelength and geometric properties. The output is evaluated in FDTD and if the results violate user set constraints, alternative solutions are calculated that fall within the set constraints.

Figure 1 provides a comprehensive schematic of the aggregate neural network framework. We divide the framework into four major subcategories: FDTD simulations, surrogate network, inverse neural network, and post-processing. As visualized in Fig. 1, the general process flow is FDTD simulations are used to train the surrogate neural network and the surrogate is used to make large-scale predictions derived from a library of materials. The predictions are then used to train an inverse neural network component. The input of the inverse network is a desired optical spectrum (λ, ε, R, T) and the output is the predicted micropyramid base span, height, substrate thickness (X_span_, Z_span_, and t_sub_), and a vector of complex refractive index values (n(λ), k(λ)) that correspond to the input wavelength. The post-processing module then interprets the output. Here, user constraints—such as the maximum aspect ratio—are used to adjust the predicted output and provide appropriate new solutions that satisfy the restrictions. The adjusted solutions are then passed through both FDTD and the surrogate model. Based on two metrics—the error between the desired input and the constrained output, and the error between the surrogate and FDTD outputs—a decision is made to either retrain the surrogate with the new simulation data, or to stop the loop if the solution is deemed to be sufficient and accurate. Any details not discussed in any of the major subcategories for all modules and connections can be found in the methods section, the supplementary materials, or in the linked code repository.

### FDTD simulations

**Figure 2:**
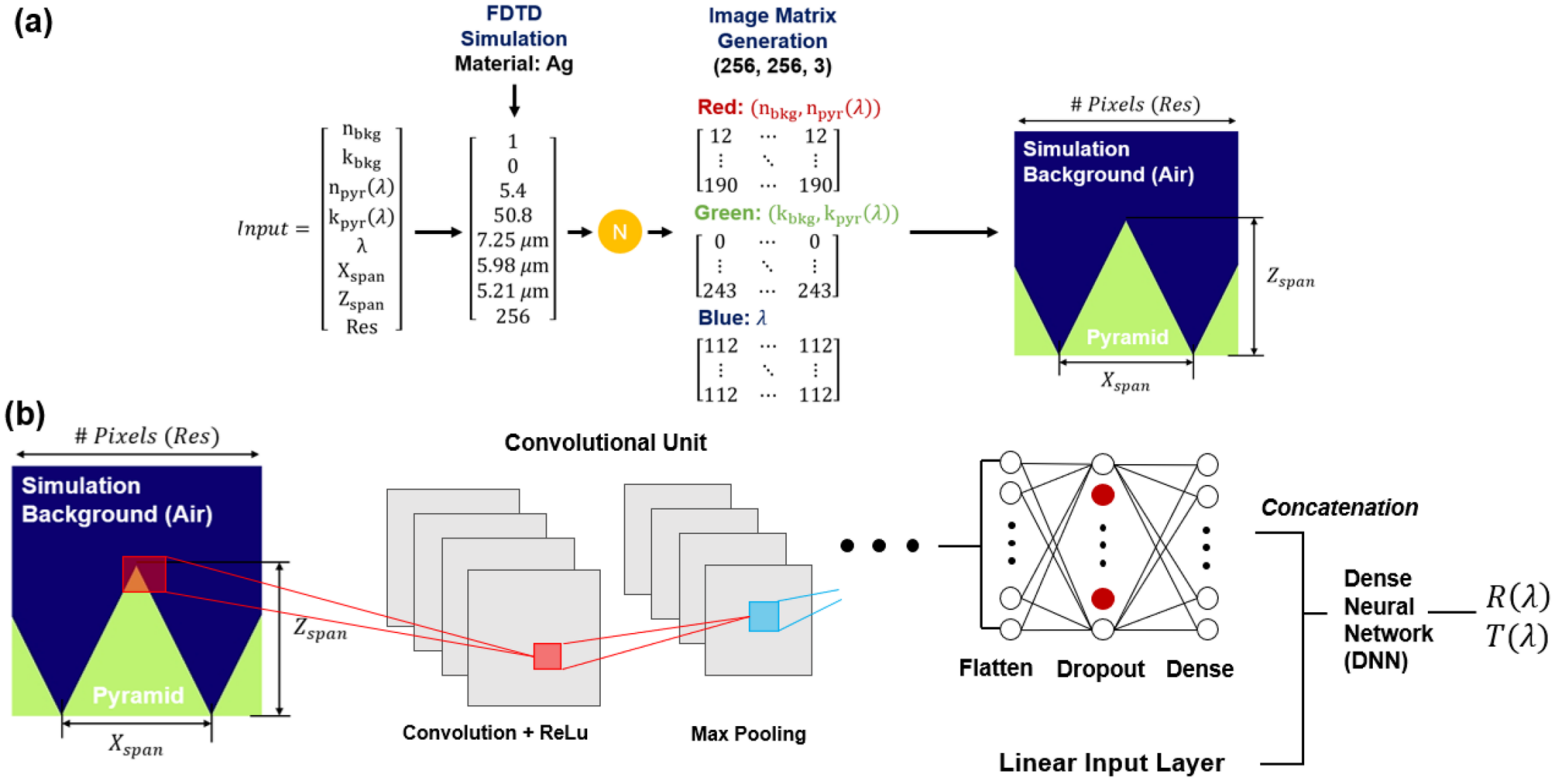
(**a**) Images for the convolutional neural network are formulated using the wavelength dependent material data. Each image contains information for a singular wavelength point. (**b**) Diagram of the convolutional neural network process for predicting material dependent optical properties from the generated images.

Solutions using the FDTD method, while accurate, can be time intensive—this is especially true for large or geometrically complex structures. For this work, the training, validation, and testing data used by the surrogate neural network is compiled from simulations completed in Lumerical’s commercially available 2D/3D FDTD solver. The simulation framework provides exact solutions for Maxwell’s equations across a finite element mesh, and the absorption and dispersion are derived from the resulting electrical fields^28,74^. Rather than simulating a 3-dimensional (3D) pyramid microstructure, we simulate the middle-cross section in 2-dimensions (2D) to minimize simulation time and to enable the generation of large quantities of simulation data. While this does lead to an overestimation of the micropyramid’s emissivity^20^ compared to the 3D micropyramid simulations, the results are still accurate as we do not vary the incidence angle in our simulations and assume the broadband wavelength source to be at a normal angle to the material’s surface. Additionally, while we could choose to use a semi-analytical approach like RCWA to run the simulations^75^ to estimate the optical properties of a 2D structure, FDTD’s accuracy, scalability, and its applicability to other more complex geometries make it a far viable long-term solution. The simulations are based upon a micropyramid geometry visualized in Fig. 2, with the key independent geometric parameters being the triangle base span (x_span_), height (z_span_), and substrate thickness (t_sub_). For this work, we assume that Kirchhoff’s law is valid and the emissivity can be derived from α = ε = 1 – R – T, where reflectivity (R) and transmissivity (T) are calculated from power monitors above and below and domain respectively and where absorptivity (α) is synonymous with emissivity (ε)^72^. To develop the simulation datasets used to train the surrogate, we generate and simulate matrices of randomly generated uniformly values for the x_span,_ z_span_, and t_sub_ for each material included. For simplicity, we assume no additional coating materials, hierarchical structures or surface roughness. Additional details on our FDTD simulation methodology can be found in both the methods section and in our prior work^2,11,20,32^.

### Surrogate neural network

Deep learning modules have been shown to be exceptionally strong and versatile in solving the so called “forward” problem^36,52^. In this case, the problem to solve is the optical response from a geometric and material input for a uniform periodic micropyramid surface. The design intent of the surrogate neural network is to act as an ultra-fast and accurate predictor of optical properties such that we can rapidly and accurately predict the optical properties of vast quantities of simulations. Furthermore, it is important that the surrogate network can extrapolate optical properties for materials beyond its original training scope. As such, we compare two methods that serve this function: an improved version of a previously developed deep-neural network^72^, and an image-based deep convolutional neural network (DCNN). Both methods utilize datasets generated in FDTD that are subdivided into training, validation, and test datasets.

#### Surrogate neural network: deep neural network

The architecture of the deep neural network—visualized in Fig. 1 of our previous work^72^ and in the supplementary materials—is designed to emulate the critical simulation inputs that influence the computed optical properties. The network employs a total of 8 input neurons: 3 geometric inputs (x_span_, z_span_, t_sub_), the source wavelength (λ), and 4 material inputs (n, k, ε_real_, ε_im_). The substrate thickness is a key geometric parameter to consider as it enables the model to interpret the relationship between the spectral optical properties and underlying material thickness and ultimately to predict the broadband spectral behavior of transmissive materials. Micropyramids of different materials are differentiated using discrete material inputs for the complex refractive index (n and k) and the correlated permittivity values (ε_real_, ε_im_). Compared to using only n and k, using both the complex refractive index and the permittivity is essential to accurately extrapolate the optical properties of materials not seen in the training process. The source wavelength (or frequency) is the fundamental factor that links the geometric and material inputs. For each FDTD simulation, we simulate 100 wavelength points (100 frequency points), each of which has a discrete solution for reflectivity and transmissivity. Accordingly, each simulation is divided into 100 discrete input vectors as the solution to Maxwell’s equations is not sequentially dependent. To strengthen the connection between the input and the output optical properties (R, T) and the key independent parameter (λ), we utilize two smaller multi-layer perceptron groupings (MLPs) to separately consider the relationship between the geometry/wavelength and the material data/wavelength. The MLPs outputs are concatenated and fed into a larger deep neural network, the output of which is a reflectivity and transmissivity value.

The DNN method is effective at being both quick to predict and in making accurate predictions, even when extrapolating. The DNN surrogate neural network has a mean absolute error (MAE) and mean-squared error (MSE) between the simulation data and predictions of 0.0033 and 1.35e-4 respectively for the “test” dataset—data that is held back from the training/validation process. As the network’s design is not limited by constraints in material input, a fundamental evaluation of the surrogate’s performance is in the prediction of the optical properties of microstructures made of materials that are outside of the scope of training. Thus, we evaluate the network on two large (1500 simulation) “unseen” datasets Al2O3/Ti, and on 100 simulations of 25 other materials in a “library”. When the simulations from these datasets are completely unseen by the training/validation process, the DNN yields an MAE between prediction and simulation of 0.0175, 0.0131, and 0.0279 for the Ti, Al2O3, and Material library datasets respectively. As the optical properties are already on a scale of 0 to 1, these errors indicate an exceptional degree of prediction accuracy when extrapolating for new materials. To improve the prediction accuracy when extrapolating, the model benefits from small “calibration” datasets. By including 5–10 simulations from the “unseen” datasets (< 1%) to the training/validation process of the surrogate, we reduce the prediction MAE to 0.0073, 0.0049, and 0.0118 for the Ti, Al2O3, and material library datasets respectively. The included simulations represent an almost insignificant number of simulations when compared to the original training and validation dataset (< 0.05%). Despite this, the inclusion has a dramatic impact on the remainder of the extrapolated data, indicating the model has strong physical understanding and only several simulations are needed to “calibrate” the model to new material behavior. In addition to the observed accuracy, the model can make predictions exceedingly fast– with over 1 million individual input sets per minute.

#### Surrogate neural network: convolutional deep neural network (CDNN)

The architecture of a second proposed surrogate method based on image processing is shown in Fig. 2. Here, we enhance our neural network design philosophy of mimicking the FDTD optical solver by making a network that analyzes a pseudo-mesh. In FDTD, the optical solution for a given combination of material and geometry is derived from solving Maxwell’s equations across a discretized mesh^76^. The only way the model can differentiate between two distinct materials (e.g., air and the pyramid) is by assigning the λ-dependent material properties to each cell. Here, we approximate that process by generating an image that utilizes the spectrally dependent material and geometric information.

An image is effectively just a tensor—as shown in Fig. 2, we take a three-dimensional matrix of material information and translate it to a standard RGB image, with each pixel containing a vector of material data. While the convolutional process is compatible with higher or lower order tensors, for ease of use and to simplify data storage/the image generator, we utilize a standard 3-channel color image. The vector used to generate each image is the same 8-input vector described in the previous section, with two additional static background material properties (n_bkg_ = 1 and k_bkg_ = 0). As the maximum Xspan and Zspan in the simulations are fixed to a maximum of 10 μm, each image is set to be an effective 10 × 10 μm—with the vertical and horizontal pixel resolution defining the “cell” length. To minimize memory consumption, we employ a 256 × 256-pixel configuration. This effectively means that each pixel represents ~ 40 nm, indicating that the minimum feature size we can effectively depict is ~ 40 nm. Thus, we eliminate any simulations with a pyramid base size or height less than 40 nm. As visualized in Fig. 2, we build pyramids symmetrically about the center of the image, filling in the remainder of the space symmetrically until the combined pyramid base span is 10 um. As an example, a pyramid with a base span of 10 um will perfectly fill the bottom horizontal axis of the image. A pyramid with a base span of 1 um will be replicated a total of 10 times in the image.

Two set of inputs CDNN architecture evaluates two inputs: the generated image and the 8-input vector of geometric, material, and wavelength information. The image component is interpreted by a convolutional neural network. The convolutional neural network is comprised of multiple “units”—each “unit” contains a convolutional layer with a ReLU activation function (defined by Eq. 1) followed by a max-pooling layer.

1 $$f\left( x \right) = \left\{ {\begin{array}{*{20}l} 0 \hfill & {for\; x < 0} \hfill \\ x \hfill & {for\; x \ge 0} \hfill \\ \end{array} } \right.$$

After several convolutions with different filter configurations, we flatten the output and apply a dropout before a dense layer to limit overfitting. The second component, the 8-input vector is used as an input to a deep neural network. This input vector contains details that either cannot be in the image—such as the thickness and excluded material properties—or information present in the image such as the material information and geometry to reinforce the model’s interpretation and prediction performance. The output of this DNN is concatenated with the output of the CNN, and then passed through a final set of dense hidden layers. The model’s output is the same as the DNN’s output—the reflection and transmission value. Precise network design details are in the methods section.

This surrogate method is more effective at accurately extrapolating optical properties for new materials when compared to the DNN only surrogate method. When only 20% of the available simulation data is used in the training/validation/testing process, we can match or exceed the performance of the DNN. The precise performance is dependent upon the selection of the material properties used in the three available pixel matrix dimensions. While the selection of the first two-pixel dimensions (the complex refractive index) is straightforward, the third quantity was a point of study. In Table 1, we show the performance of the CDNN architecture in predicting the Ti, Al2O3, and material library datasets when different quantities are used in the third matrix dimension. Based on these results, we observe that the wavelength is the most effective parameter to use in the third dimension. This provides additional confirmation that the wavelength is an extremely important parameter in enabling the model to build proper connections between the input and output. The model evaluations shown in Table 1 are performed with the complete unseen datasets, we apply the 20% limitation only to the model’s training data.

**Table 1:** Comparison of training, validation, and unseen material dataset performance for the DNN and CDNN methods.

Method (% Data)	Channels	Train	Validation	Ti	Al2O3	Library
DNN (100%)	N/A	0.0032	0.0033	0.0175	0.0141	0.0279
CDNN (20%)	n, k, 0	0.0031	0.0036	0.0171	0.0225	0.0373
CDNN (20%)	n, k, ε_im_	0.0019	0.0032	0.0266	0.0176	0.0378
CDNN (20%)	n, k, ε_real_	0.0029	0.0031	0.0233	0.0184	0.0346
CDNN (20%)	n, k, λ	0.0025	0.0031	0.0185	0.0163	0.0314

To greatly speed up the training/validation time, we only use 20% of the available data to train the CDNN (~ 710,000 images of 3.55 million).

When we enable the model to see the full simulation dataset in training that the DNN does (3.55 input vectors, or 3.55 million images taken from 35,500 simulations), the CDNN method significantly outperforms the DNN method. The Ti, Al2O3, and library datasets have an evaluated MAE of 0.0155, 0.0113, and 0.0226 respectively. When we calibrate the model with 10 simulations as previously demonstrated in the DNN, this decreases to 0.0067, 0.0043, and 0.0098 respectively. Despite being more accurate than the DNN, due to the relative increase in parameters and memory scale, the training time and prediction time for the CDNN is significantly longer than the DNN.

### Inverse neural network

We harness the rapid prediction capabilities of the surrogate network to iteratively train a neural network that solves the inverse design problem. That is, we invert the forward problem to predict what material and microstructure geometry will best match a desired system optical response. The input of this network is the spectrally dependent reflectivity, transmissivity, and emissivity corresponding to a desired wavelength range.

The architecture of the inverse neural network, as depicted in Fig. 1, solves the inverse problem by considering the entire spectral distribution. The network input (400 × 1) is a vertically stacked combination of the predicted reflectivity, transmissivity, derived emissivity (ε = 1 – R – T), and the wavelength vector the optical properties are sequenced to. Correspondingly, the inverse network output is a vertically stacked combination of the geometric input and wavelength dependent material properties (n, k, ε_real_, ε_im_, 403 × 1) as visualized in Fig. 1. Unlike the surrogate network, we cannot separate the inputs of an inverse network into single input vectors. An “inverted” solution for a single set of wavelength dependent optical properties has an unbounded number of potential solutions, so to design an effective inverted network the input must be the entire sequence. In our first design iterations of the deep neural network surrogate, we considered using the entire sequence of wavelengths/material data as an input and reflectivity/transmissivity as the output. While this method was effective, because Maxwell’s field equations are not sequentially dependent, the surrogate solver was much more effective when the sequences were broken up and individual vectors based on a single wavelength point were used as an input. This method also dramatically expands the scope of the training set from 35,500 simulations to 3.55 million input sets, making a limited number of simulations more effective in developing a surrogate with physical insight that can solve the forward problem more accurately. Once the surrogate is trained and can produce accurate results, however, the number of simulations becomes trivial, as we can effectively estimate the solutions to 10,000 FDTD simulations (with 100 wavelength points each) in approximately 60 s using the surrogate network.

To generate training data for the reverse neural network, we pass in large grids of data to the surrogate network for prediction and collate the output into discrete input and output sets. For each material in a library, we generate a grid of 200 × 200 geometric combinations. These combinations are formed by meshing linearly spaced vectors for the X_span_ and Z_span_. The minimum and maximum values for these vectors are based on the minimum and maximum observed value of X_span_ and Z_span_ in the surrogate’s training dataset. In total, the grid has 40,000 geometric combinations (or 40,000 simulations) for a single material. For each geometric combination we attach a wavelength vector of length 100, leading to a sum of 4 million inputs per material that are passed to the surrogate. While the wavelength vector attached to each geometric combination was originally a linearly spaced vector that ranged from λ = 0.3 um to λ = 16 um, we found that using a linearly spaced wavelength vector with randomized min/max values for each geometric combination increased the versatility of the training dataset and thereby increased the robustness of the inverse neural network. All generated grid data is normalized before being passed into the surrogate network for prediction. The final non-material parameter—the substrate thickness—is also randomized via a uniform random generation process. Details on the random generation process for the substrate thickness and wavelength vector can be found in the methods section. This grid generation process is repeated across all materials in a material library. The material library contains 50 materials: a list of the materials and their references are provided in the supplementary document. The number of materials in the library is easily scalable and are a non-exhaustive representation of material properties available for a microstructure. In total, we use the surrogate network to estimate 2 million simulations, or 200 million sets of inputs. We then sequence the predicted optical properties using the wavelength vector (of length 100) for each simulation.

The inverse network contains three distinct neural network components that are designed to work in tandem to extrapolate a geometry and material that best fit the desired optical response. The first of the components is a deep neural network consisting of multiple hidden layers that directly take in the (400 × 1) input vector. On a rudimentary level, simply inverting the surrogate’s DNN structure—but with the progression of wavelengths instead of an individual wavelength point—could be effective. Through our development process, however, we discovered that this more simplistic approach lacked physical insight and would often result in a non-physically viable output. Although the solutions to Maxwell’s equations for a given wavelength, material, and geometry—the problem the forward network addresses—are not sequentially dependent, abrupt changes or singularities in material properties across a spectrum are seldom. Thus, developing insight on the relationship between a sequence of optical inputs and material properties is crucial in building a physically grounded model. To address this, we remap the linear sequence of optical properties into a “time”-dependent matrix and use it as an input to a recurrent neural network (RNN). That is, we map the 400 × 1 vector of (λ, ε, R, T) into a 1 × 100 × 4 matrix (λ, ε(λ), R(λ), T(λ)). We select bi-directional long-short term memory (LSTM) layers as the constituent component to the RNN. LSTM networks are more effective than other RNN methods for long-range dependencies in data^77^, and the bi-directional attributes enables the network to learn both dependencies in the forward and reverse direction. Additionally, we utilize dropout layers between LSTM layers in conjunction with L2 regularization to reduce overfitting. The outputs of the RNN and DNN components are then combined using a matrix multiplication and fed into a third component, another DNN. As opposed to directly linking the network output to the final DNN/RNN layers, a DNN between these two networks and the final network output facilitates an additional layer of non-linear abstraction and learning from the outputs of the two preceding neural network components.

**Figure 3:**
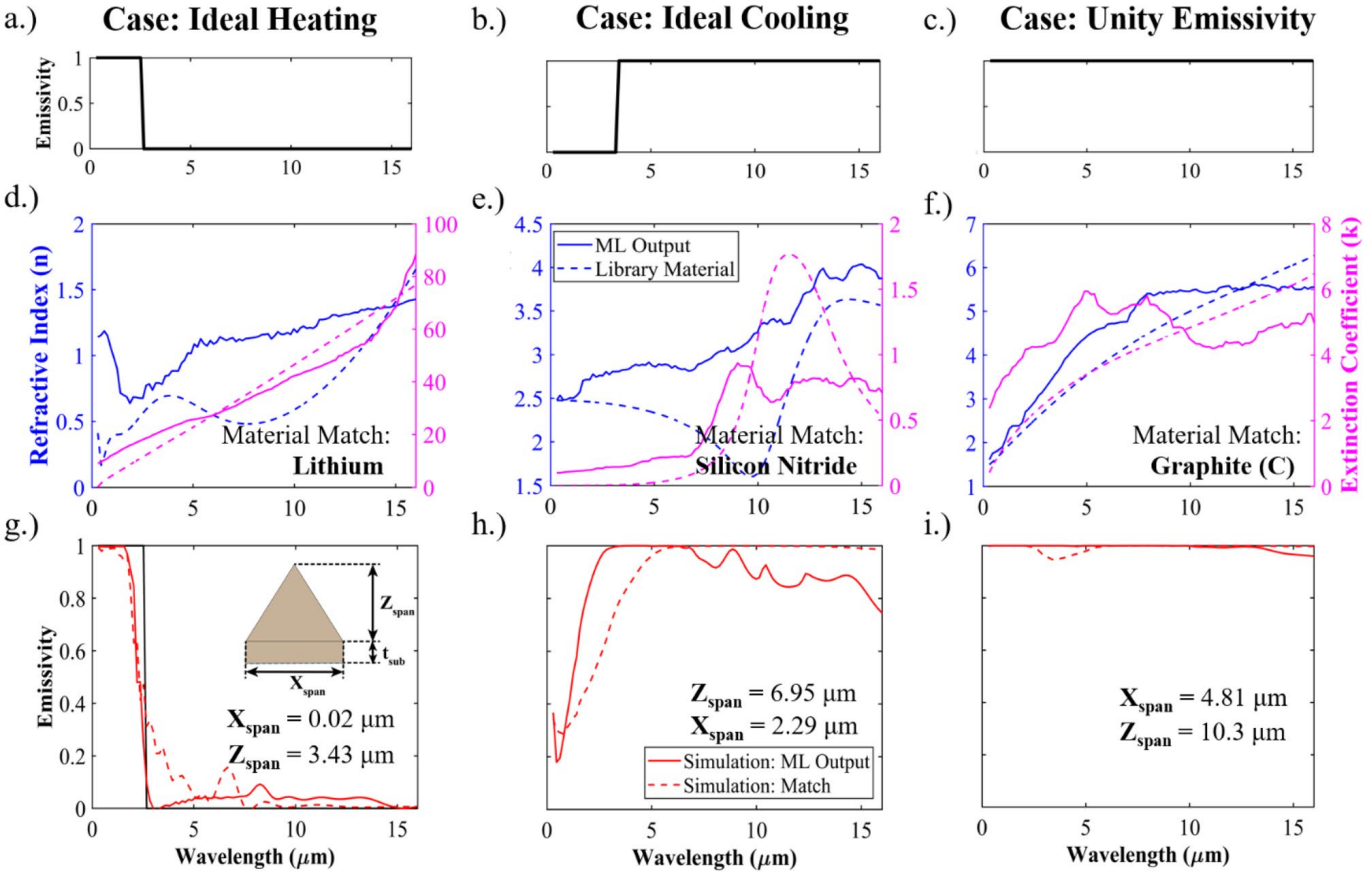
(**a**–**c**) Emissivity spectrums of three test cases (Ideal heating, ideal cooling, and unity emissivity) input into the inverse spectrum. The reflectivity is computed as R = 1 – E, and transmissivity is set to 0. (**d**–**f**) ML generated refractive index (n) and extinction coefficient (k) for each of the test cases compared to the material properties of a material in the library that most closely matches it. (**g**–**i**) FDTD simulation results for both the ML generated material and the closest matching library material.

Figure 3 shows the output of the inverse neural network for several broadband test inputs and results once the network outputs are simulated in FDTD. We utilize three thermally relevant test spectrums as a baseline evaluator of the inverse network—unity emissivity, an ideal heating emission spectrum, and an ideal cooling spectrum. These emission spectrums are shown in Fig. 3g–i. For these test cases, we set the spectral transmission to be 0 and R = 1 – ε. In Fig. 3a–c we compare the material properties predicted by the neural network to the material properties of a material in the library that best matches it. The predicted geometric conditions are given in Table 2. For all cases, the projected material properties have a close match in the library. In Fig. 3d–e we compare the results of FDTD simulations using the network generated material and the closest match material for the same predicted optimal geometry. For both the heating and unity case, we observe that the neural network generated material outperforms the library material. Additionally, we note that both the generated material and the library material produce a result that matches the desired input to a high degree of accuracy. This is despite the input having a non-physical step-function behavior. The ideal cooling spectrum (Fig. 3i) has a larger departure between the desired spectrum and the true outcome for both the ML and library generated materials. The observed error is attributed to the physical limitation of material properties and the imposition of zero spectral transmission. This assumption is outside of usual physical intuition for the ideal cooling case, where due to the physical material limitations, most emissive materials (e.g., TiO_2_, Al_2_O_3_, PDMS, etc.) in the infrared are transmissive in the ultraviolet (UV) to NIR wavelengths. Thus, this represents a design challenge for a single material to perform both functions, and the inverse neural network attempts to abstract a physically bounded material that fits zero spectral transmission. The identified properties match well across the broader spectrum but do not capture the intended performance in the visible to near infrared regions (λ = 0–4 um). If we allow transmission in this region, we receive an expected output of PDMS, details for which can be found in the supplementary document.

**Table 2:** Library material that most closely matches the ML generated material output n and k in addition to the predicted geometric parameters.

Case	Closest material match	Xspan (um)	Zspan (um)
Unity emissivity	C (Graphite)	4.81	10.3
Ideal heating	Li	0.02	3.43
Ideal cooling	Si_3_N_4_	2.29	6.95

**Figure 4:**
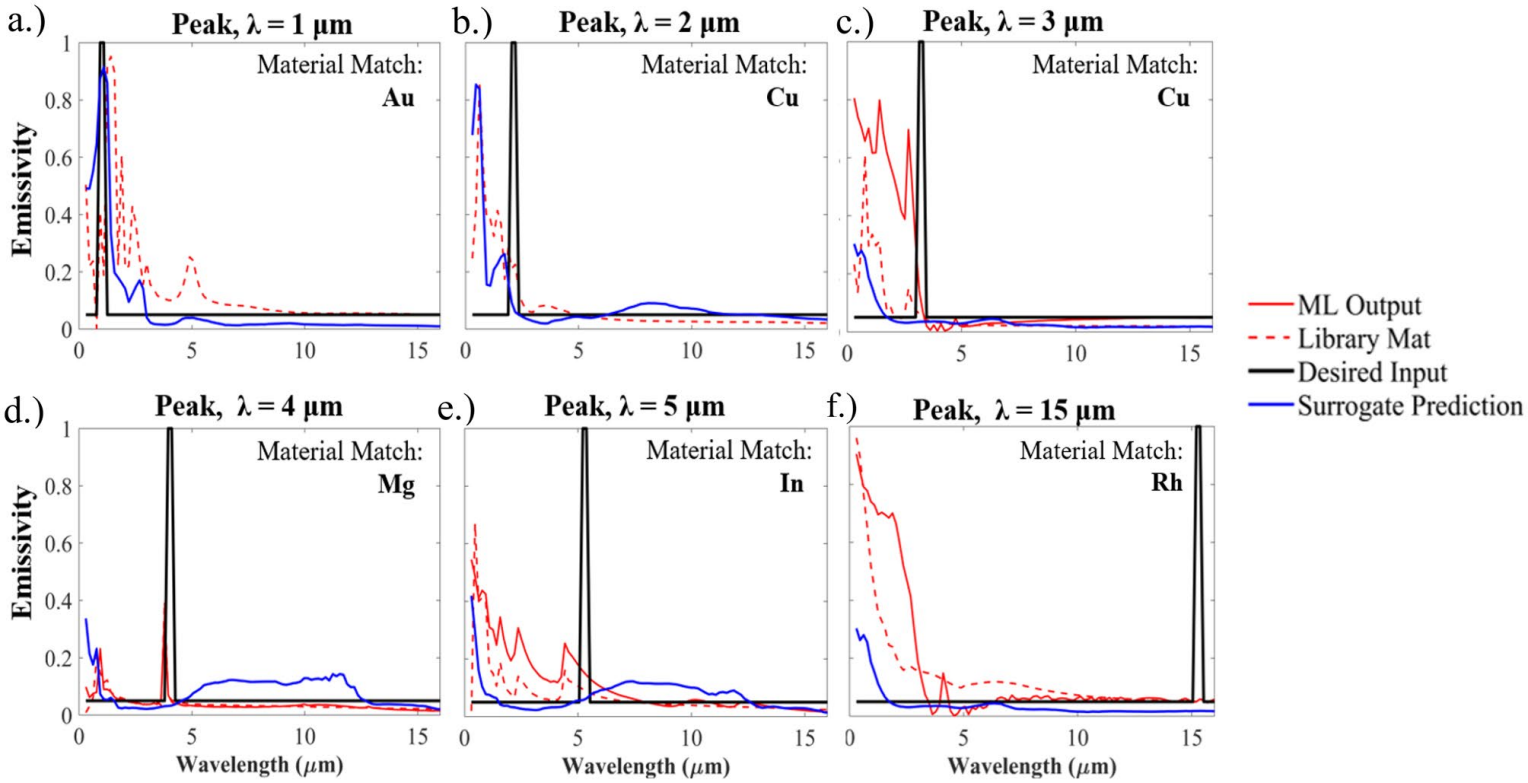
(**a**–**f**) Narrowband simulation results using the inverse neural network. The input spectrum has an emissivity of 0.05 throughout the λ = 0.3 to 16 um, except for two points that define the “peak” location which have an emissivity of 1. The reflectivity is defined by E = 1 – R and the transmission is set to 0. The results are compared to the desired input as well as the surrogate predictions for the same material.

The inverse neural network is not limited to broadband design. In Fig. 4, we show how the inverse neural network can be applied to narrowband microstructure design. In this case, we define narrowband as 2 emissivity points with a unity value around the intended wavelength peak. The inverse neural network results for 6 different wavelength points (1, 2, 3, 4, 5, and 15 um) are shown in Fig. 4a–f. The design outcome highlights both the strengths and weaknesses of the presented inverse neural network methodology. The geometric design space is limited to the relatively simple micropyramid geometry and can only utilize one material. Thus, with the implemented neural network architecture, our model stays within constrained and physical material behavior, attempting to find valid solutions without creating a completely arbitrary material. This leads to valid narrowband solutions in the low wavelength regions where geometry can be attenuated to generate plasmonic resonance and resonant behavior. This behavior is particularly evident in the solutions visualized in Fig. 4c,d, with peaks at or near the desired location, albeit with either reduced performance or peaks beyond the desired location. The neural network can readily identify solutions that are physically feasible, but is challenged to find resonant behavior that results in narrowband solutions for the mid-infrared wavelengths. These plots result from the both the physical limitations imposed by the input, the training data available to the network, and the fundamental physics of the micropyramid system. Despite these challenges, the inverse network still can be shown to identify physical behavior outside of the scope of its training data. In Fig. 4d, the surrogate model’s predictions do not indicate resonant narrowband behavior at 4 um, but when simulated the inverse neural network output shows a significant degree of narrowband performance. This indicates that the reverse neural network can abstract solutions beyond the training data and identify behavior that the surrogate cannot, but the network is still constrained by the fundamental physics.

### Post-processing: constraints and solution viability

While the inverse neural network output can accurately predict material and geometric properties that result in the desired optical spectrum, a key limitation of our open-ended neural network architecture is that it cannot directly accommodate design constraints. This presents a significant challenge in making the inverse design process functional. Ideally, our aggregated network will output a solution that is translatable to a fabricated surface morphology. This challenge is evident from the geometric results for the ideal heating case in Table 2; the aspect ratio of the ML predicted structure is ~ 400 (Zspan/Xspan), which is clearly an impractical microstructure. To address this, we take the output of the neural network and use a post-processing methodology to provide new solutions that fit within user set constraints. Though it is possible to constrain the neural network itself via methods such as custom activation functions on the output neurons, limiting the input dataset, or introducing limits in the input, we choose to post-process the neural network output to maintain a robust inverse solver. For this work, we focus on constraining the aspect ratio as it plays a key role in determining if a microstructure is manufacturable. Other constraints, such as a material’s maximum temperature, thickness limitations, etc., are important and can be easily incorporated for more advanced design optimization.

The post-processing methodology has several stages: inverse prediction, material matching, geometric adjustment, surrogate prediction, simulation, and finally output comparison. Precise details for all the stages of the post-processing method are provided in the methods section. The first stage is to take an optical spectrum, pass it through the inverse network, and output a set of geometric properties and spectral material information. From there, the ML generated material data is compared to existing materials in the material library. We then adjust the ML generated geometry to align with the set maximum aspect ratio. Using the adjusted geometry, we randomly select new constrained/viable geometries and simulate them using the surrogate; the most optimal solutions are passed to FDTD. The post-processing method then compares the “ground-truth” FDTD to both the surrogate output and the desired input. This process is performed for both the ML-generated material and “best-fit” library material and identifies a constrained geometry that is optimal for both the ML-generated material and selected library material.

**Figure 5:**
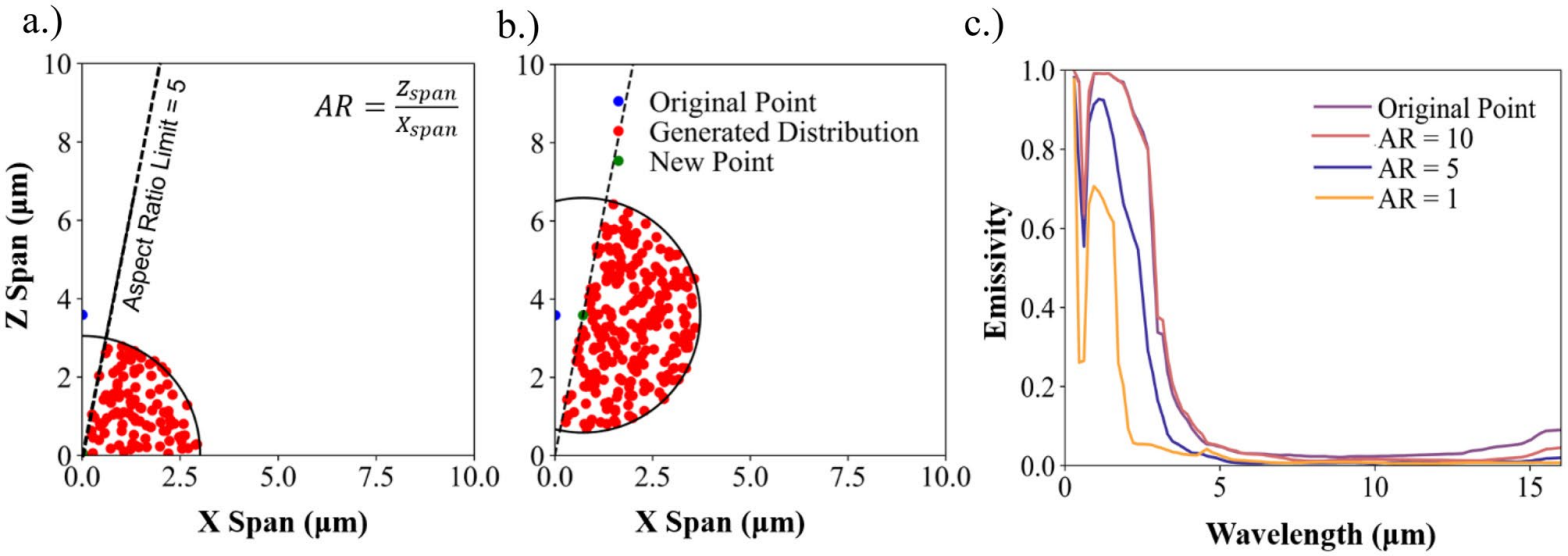
(**a**) Example of vertical and (**b**) horizontal reorientation of inverse ML output and subsequent generation of randomly distributed solution points about the adjusted geometric solution. Example shown is using an aspect ratio limitation of 5. These solutions are evaluated using both FDTD and the surrogate, the most optimal new solution is using a process described in the methods section. (**c**) The identified optimal point at each aspect ratio is shown for each aspect ratio. While the solution is not as optimal as the original ML generated geometry, the we can still identify geometric designs with exceptional performance.

Figure 5a,b shows the post-processing method’s application to select a new viable solution for the ideal heating case discussed above. For this demonstration, we show solutions when the aspect ratio (Z/X) is limited to 10, 5, and 1. The new geometric solutions are simulated using the surrogate, and the results are compared to the predictions for the unconstrained ML generated geometry. Compared to the desired input spectrum, the constrained cases have an LSE value of 1.229, 1.396, and 1.567 for AR = 10, 5, and 1 respectively. It is evident that while the results decrease in adherence to the desired spectrum as the aspect ratio is limited, it is also evident from Fig. 5c that the adjustments to the geometry to accommodate the limited aspect ratio constraint still yield highly optimal results. It is evident that these solutions deviate from the global maximum but are still highly effective when constrained. If the constrained solution is deemed to not be viable enough, additional materials can be included in the search, forming a more advanced material matching algorithm than previously utilized^20,72^.

### Aggregated neural network

By combining the individual components, we form the aggregated system loop visualized in Fig. 1. The aggregated system is designed in a way that it can learn from previous mistakes and enhance its capabilities and accuracy over subsequent generations. Despite the accuracy of the image-based surrogate, especially for unseen materials, the time required to train and retrain the image-based convolutional network compared to the simpler deep neural network surrogate eliminates it as an option for the aggregate system. The geometric/wavelength simulation grid data generated for each material is passed into the DNN surrogate and the ensuing predictions are the basis of the inverse network training data. If changes are made to the surrogate or more materials are included in the library, the grid data will be regenerated. We then employ the broadband and narrowband test cases in Figs. 3 and 4 to test the performance of the inverse network in making predictions. If either significant deviations between the desired result and actual result are encountered or the surrogate prediction deviates significantly from the FDTD results, we perform additional simulations via our post-processing solution generation method. These results are incorporated in the training data for the surrogate, and the surrogate/inverse network are retrained. Post-processing then serves an additional function as a pseudo-adversarial checkpoint where the generated results are compared to true results and if there is an undesired outcome the network is retrained with new simulation information.

## Discussion

A core challenge facing the design of microstructures is the time required to simulate and optimize a design. For the first component, in stark contrast to an FDTD solver, a neural network can make predictions comparatively instantaneously. The generation of the ~ 40,000 FDTD simulations used to train, test, and evaluate our surrogate network required months of computational time, whereas the surrogate network can predict a 40,000-simulation grid (4 million sets) in approximately 4–5 min. The DNN surrogate network can predict a 100-wavelength point simulation in approximately 6 ms, representing a 4 to 6 order of magnitude speed increase compared to using the FDTD solver for the same task. Considering the demonstrated accuracy of the network across a library of materials, this method is highly effective in substantially reducing the need for complex optical solvers to estimate spectral optical properties across a large material library. As previously demonstrated, once the model has a connection to the physics of the problem, very few simulations are required to “calibrate” the model to new materials. Our DNN surrogate only requires an average of 5–20 FDTD simulations to be included in training per material to bring the prediction error for that material to near to the rest of the dataset.

A fundamental advantage of the surrogate network compared to FDTD simulations—speed—is leveraged in this work to build a comprehensive dataset for training an inverse solver that aims to address key challenges in design optimization. In our previous publications, we addressed optimization using a thermal figure of merit and a “brute force” optimization method. That is, we designed an objective function to describe a desired spectrum and use the FDTD or surrogate optical output to solve the thermal equations and then use the objective function to determine which spectrum most optimally matches the input. This method, clearly, is incredibly slow and cumbersome. Using a neural network to perform the inverse task—much like the forward solving network—is orders of magnitude faster than this approach. The speed difference relies on the fact that the inverse neural network can learn the pattern between the optical properties and material/geometric properties, and directly take in a spectrum and output a material/geometry that suits it. Rather than a roundabout methodology that relies on identifying the best spectrum using grids of surrogate generated data across multiple materials, the inverse network trains on those grids and provides a user with a nearly instantaneous output to a selected spectrum. This method also opens the door to non-intuitive solutions as we can potentially identify new materials that are not in the material library.

Determining the appropriate surrogate method to generate the training data for the inverse data is an important consideration in the design of the aggregate system. Ultimately, due to the large grids of data used to generate the training data and the training/retraining process, the inverse network necessitates a fast surrogate. The DNN methodology, as compared to the CNN, is significantly faster in making predictions and in training. When trained on the full dataset, the image-based approach can require hundreds of hours to train using our computational resources. Comparatively, the DNN can train on the entire dataset in approximately 25–50 h. Additionally, the prediction time is significantly longer for the CDNN, making the generation of simulation data to train the inverse network far less efficient. While the CDNN method is more accurate, the incremental accuracy increase we observe does not justify the time cost in this case. We could reduce the size of the images used by the CDNN to speed up the network training and prediction times, but then we would lose spatial resolution and must eliminate a greater quantity of simulations from consideration. The efficacy of the CDNN will be more apparent in future work that relies on more complex structures. The images used as an input are not specifically bound to a single geometry, and a CDNN surrogate could be constructed that solves the optical properties of different geometries or even abstract shapes. This could also include multiple geometries, hierarchies, or even structures that include multiple materials such as coated structures or composite structures. In addition to these options, a CDNN could also be converted into a tandem inverse solver with a generative network. Ultimately, while the micropyramid geometry we show in the present work is relatively simple and does not necessitate the image-based surrogate to train the inverse network, potential future options for complex inverse neural networks based on an “image derived mesh” are boundless.

The primary strength of the inverse neural network design—its ability to generate a unique set of spectral material properties—leads to significant design and implementation challenges. A core concern in the design of the network is determining how to balance a desire to remain physically bounded while also enabling the model to find ways to extrapolate in new and unique ways. A simple approach would have been to simply one-hot encode material classifications, thereby eliminating non-physical material outputs. This approach is undesirable as it not only eliminates the ability to extrapolate new materials, but it also significantly reduces the ability to explore different materials in post-processing. As demonstrated, the model generated material properties can outperform the materials in the library. As the scope of the surrogate and inverse network continue to expand, more materials are added, and more simulations are performed, we expect that the inverse network will not only increasingly outperform existing materials but could be used to identify “effective” refractive indices and inform the reverse engineering of material combinations that match it. These strengths are lost with rigid material classification, and to maintain a robust solver we designed the network to accommodate material outputs that are only bounded by the arbitrary list of materials used to generate the training data.

The design choice to allow the network to choose discrete material properties—as opposed to classification—does lead to significant challenges and necessitated design compromises. Early iterations of the inverse network design utilized a single DNN with a single vector (400 × 1) input, but we found that the output would often be non-physical or unrealistic. Thus, to ground the model from pure abstraction of material properties, we implemented a sequential method utilizing an RNN to ensure that the model could learn the forward and backward relationships of the material properties. This enables the model to have a certain level of physical insight into how materials usually look, so that when it generates the output the output should resemble, but hopefully surpass, the performance of microstructure made of the “best-match” material in the library. Additionally, our network’s early construction only considered the emissivity as an input. Over subsequent model iterations, however, we found that the inclusion of the two parameters that determine the emissivity—the reflectivity and transmission—enabled not only more control for the user, but also additional reinforcement in the model’s ability to abstract physical relationships between the material properties and input spectrum.

An important consideration is the impact that geometry has on the optical properties and thereby the thermal performance compared to an untextured surface. The materials generated and identified through the neural network process conform to physical intuition for each of the test inputs. While this intuition may be sufficient for a material matching algorithm, the application of optimized texture further enhances absorption and improves the thermal and optical outcome for a system. This is particularly true for the Ideal heating input shown in Fig. 3a, where optimal texturing leads to a surface that can absorb over 96% of incident solar radiation, compared to approximately 15% when the surface is untextured. While untextured (completely smooth) graphite of < 100 um thickness has an emission efficiency of ~ 33.5%, ML identified optimal texturing increases the emission efficiency to ~ 99.6%. Texture has a more minimal impact on Si_3_N_4_, raising the emission efficiency from ~ 75.6–99.9%. The derivation and graphical representation of these values is shown in the supporting materials.

Several key issues arise from the selected network design. The first is in identifying and subsequently correcting any mistakes made by the inverse neural network. Due to the open-ended design of the material outputs and the large variability in the inputs, it is very easy for a user to specify a non-physical input that can result in the network making a valid approximation for much of the broadband spectrum but missing a key narrow portion of the spectrum. This is particularly apparent in the narrowband case shown in Fig. 4 where a large broadband wavelength input is used in conjunction with a non-physically intuitive spectrum for a single material microsystem. This design challenge necessitated switching from only broadband wavelength inputs (0.3–16 um) in the training data to randomizing the wavelength/material vector passed into the surrogate. Still, results still demonstrate that the model will attempt to solve the problem but of course cannot correct a user’s input. It should be acknowledged that the network is trained using physically bounded and sequential results, so abstracting a solution for what could be a non-physical desired spectrum should not be expected to have a high degree of accuracy. A second related challenge comes from the material data pathways and generation methods. The FDTD method relies on curve-fit data based on experimentally sampled measurements. Our network builds a cubic-spline fit model based on FDTD generated material data. When we want to simulate the neural network generated material properties, we need to pass it into FDTD in the same way physical measurements would be. This can lead to some fundamental challenges in curve-fitting and automation, as the FDTD curve fit for ML generated data may be completely incorrect and require manual intervention. This process also limits our options to use ML generated data in the aggregated system training loop. If the actual properties deviate from the inputs, incorporating the data could lead to significant prediction inaccuracies. For future modelling efforts with more advanced systems and multi-material composites, very careful interpretation and interpolation of material properties will be required to properly represent and predict new materials.

Another challenge is to constrain the output based on user set limits. Potentially applicable constraints are plentiful, but for this work we focused on constraining the aspect ratio as it is a crucial element in determining the manufacturability and scalability of a microstructure. We presented a solution to both key issues by introducing a post-processing module. This module is not a neural network, and it operates outside of the “black box” of neural network design and can be more easily adjusted to account for real scenarios using the optimal output provided by the inverse network. An apparent shortcoming to neural network design is in understanding the ever-increasing complexity of non-linear abstraction that occurs inside of the “black-box” of the hidden layers. While we could introduce limitations on the network, add new variables, etc., to account for the constraints, this may not only reduce the robustness of the architecture but also could make it difficult to supplement or adjust the solution with physical insight.

In effect, the post-processing module takes the role of both an adversarial checkpoint as well as a local-minimum optimization method. If the imposed constraints are violated, the post-processing module infers new solutions and determines which of these are most optimal. Of course, this method could be used in the same “brute-force” manner that we utilized in previous publications^20,32,72^ to determine a local minimum optimal solution from the surrogate, particularly if it was combined with a gradient-descent optimization method. However, this has the same issues as the previous approaches in that the result is not only likely to be a local minimum, but that we would be required to repeat the process for every material in the library. The aggregated system is designed to leverage all the modules to automatically learn and correct the networks if an incorrect prediction is made by either the surrogate or inverse network. By generating new solutions, simulating them, and then comparing and simulation results to the surrogate results and desired input, a decision can be automatically made to include the simulation data in a subsequent loop of data generation and model training. This process is directly transferable to any microsystem design, and for more advanced iterations that include additional limiting parameters such as temperature dependence and temperature dependent material properties. The unbounded nature of the entire loop also unlocks unique perspectives and solutions that would otherwise be infeasible.

## Conclusion

We have demonstrated a platform that can output discrete and unique material and geometric properties that will lead to an input optical spectrum. The models are not rigidly constrained by material classification, and the network can be used to identify the material properties that would best solve the problem. The inverse solver enables the design of a material matching algorithm that can identify what materials are best suited to match a desired optical response based on user set constraints. Furthermore, the inverse network input is not limited to a preset input wavelength vector, enabling the dynamic exploration of narrow band and limited wavelength solutions in addition to more traditional broadband inverse optimization. As a part of the platform, the exhibited post-processing method takes the output of the inverse neural network, removes it from the black box of neural network processing, and allows for adjustments to the neural network output based on set constraints. The post-processing section also serves as an adversarial node to the combined system, connecting to the FDTD simulation source and introducing targeted simulation data to improve the neural network in subsequent generations. While we only use the deep-neural network derived surrogate solver as a part of this process, the image-based method we developed could play a pivotal role in future iterations of inverse design networks for more complicated microstructures or multi-material systems that cannot be simply represented in a deep-neural network. Our methodology not only effectively replaces FDTD simulations for micropyramids, but it also enables near instantaneous inverse-design and optimization, allowing for near instantaneous complex and comprehensive design optimizations.

## Methods

### Data and code availability

The datasets and models generated and/or analyzed during the current study are available in the Inverse-Optical-Neural-Network repository, https://github.com/jmsulliv/Optical_Prediction_Reverse_Network.

### FDTD simulations

We perform FDTD simulations in Lumerical/ANSYS’s commercially available FDTD simulation software. The unit cell shown in Fig. 1 replicates the major variables simulated—x_span_, z_span_, and t_sub_. A plane wave source with normal incidence is placed in the z-direction. For this work we do not consider angular dependence of the optical properties or of the dependence of the optical properties on the polarization angle. The injection wavelength spans a linearly spaced vector of 100 wavelength points that begins with λ_min_ and ends with λ_max_. Perfectly matched layers are applied in the direction of the injection source to prevent boundary reflection at both the top and bottom of the domain and periodic boundary conditions are placed perpendicular to the wave source. Frequency-domain field and power monitors are placed above and below the PML boundary layers to monitor reflection and transmission respectively. Emissivity is computed using Kirchhoff’s Law, α = ε = 1 – R – T. The monitors are solved at every frequency/wavelength point, leading to a one-to-one matching of the simulation output to the wave source.

For the surrogate training data, while there are some variations in the wavelengths used to generate the material data^72^, the majority of the materials are simulated using a λ_min_/λ_max_ of 0.3/16 μm respectively. Vanadium Dioxide is divided into two separate materials: that of an insulation phase (ceramic behavior) and metallic phase (metallic behavior)^78^. The value of t_sub_ depends on the material selection. For metals (Ni, Al, Ag, W, Sn, Fe, Ta, Cr, Ti) and SiC we simulate over a range of random t_sub_ values confined by a minimum value of 1 μm and a maximum of 3 um. For transmissive materials with a wide range of substrate dependent performance (e.g., VO_2_, SiO_2_, PDMS, Al_2_O_3_) we choose the minimum thickness to be 1 um and the maximum to be 100 μm. For simulations that occur as part of the post-processing phase in the aggregate network loop, simulations directly take in the output properties of the post-processing module/neural network.

### Network architecture and optimization

We use a deep neural network with fully connected dense layers. Our deep learning approach is built upon the open source keras library in python^79^. The surrogate network, as previously published^72^, uses an optimized DNN with 8 fully connected dense layers with 400 neurons per layer, and both MLPs are 4 layers of 50 neurons each.

The CDNN network combines a similar DNN structure with a CNN architecture. The first DNN structure takes in the same input vector as previously discussed network but uses a smaller set of layers and neurons. The CNN uses 6 groups of convolution – ReLU – max pooling. The filter configuration for the convolutional layers is 64, 128, 256, 512, 512, 512. The final convolutional layer is followed by a max pooling, dropout (0.25), flatten, dense, dropout (0.5), and then a final dense layer. The output is concatenated with the DNN structure and fed into another DNN, which is 7 fully connected dense layers with 1024 neurons each. We utilize a custom image generator process to handle the import of images and their associated deep neural network properties into the model.

The inverse network takes the same set of inputs (1 × 400 vector) and applies it in two separate ways. The first is a direct input to a deep neural network, with a 1 × 400 input shape, which consists of 6 fully connected dense layers of 750 neurons each. The second input recasts the original 1 × 400 vector into a 1 × 100 × 4 vector and is put into a recurrent neural network. The RNN is constructed of 3 bi-directional LSTM modules—that is, a bi-directional LSTM layer followed by a dropout (0.5). Each bi-directional LSTM layer has 320 neurons, and the final layer’s output is non-sequenced. The outputs of the RNN and DNN are concatenated and then fed into a larger deep neural network that consists of 6 layers of 1000 neurons each. The final output is 403 neurons with no applied activation function. We experimented with different methods of combining the two outputs—including matrix multiplication, addition, and subtraction—but found that the concatenation was consistently yielded the best results.

For training all the models, we utilize a MSE loss function and validate/evaluate using an MAE score based on Eqs. (1) and (2) respectively, where $Y_{i}$ is the predicted value. A key change to the model training compared to prior results is that all data is made available to the network and there are no “unseen” materials in the training process. For the grid generation process, we do utilize several materials that are outside of the scope of the training process, but no simulation data was generated for these materials prior to training the reverse network. The full list of materials included in training and in grid generation are provided in the

2 $$MSE = \frac{1}{n}\mathop \sum \limits_{i = 2}^{n} \left( {Y_{i} - \widehat{{Y_{i} }}} \right)^{2}$$

3 $$MAE = \frac{{\mathop \sum \nolimits_{i = 1}^{n} \left| {Y_{i} - \widehat{{Y_{i} }}} \right|}}{n}$$

In all cases, optimization of the hyperparameters is performed with the built-in hyperband optimization method^80^. Adam is the optimization engine used for the network training in all cases. To minimize overfitting, we utilize L2 regularization in the training and validation process, in addition to utilizing early stopping, checkpoint save, and reduce learning rate on plateau callbacks with low patience values^72^. Some models incorporate dropout layers to further reduce model overfitting.

### Datasets and normalization

All datasets used by the neural networks are derived from FDTD simulation inputs and outputs directly. For each material in the training/validation/test dataset of the surrogate models, we generate a uniformly distributed random matrix for each of the geometric properties to use as inputs for the simulation. The simulation wavelength and n and k values are taken from each simulation and split into sets of input data, spanning a total of 8 neural inputs (n and k are converted into ε_real_ and ε_im_). The simulation output is 100 emissivity and 100 reflectivity points that one-to-one match the simulation wavelength vector, which is divided into pairs for each λ. The ε_real_ permittivity value is of particular concern due to the negative values induced by -k^2^ term shown in Eq. (4).

4 $$\varepsilon_{real} = n^{2} - k^{2}$$

A fundamental problem faced is that optically, the difference between k = 1e-4 and 1e-3 is not mathematically large, but the difference does have a large impact on the transmission behavior through the substrate. Thus, the data is grouped near 0 but we need to differentiate values in a meaningful way to distinguish the physical behavior of each material. Log normalization reduces the severity of the weighted inputs but does not solve it. For all of the datasets shown in this work, we utilize quantile normalization with sklearn’s built in quantile transformer, to generate a uniform distribution of inputs for k, t_sub_, ε_real,_ and ε_im_. A change from our previous results^72^ is that we simplify the normalization pipeline by normalizing the refractive index n and the geometric properties using the quantile method. All datasets used in this work, and the techniques used to normalize, denormalize, and configure the data are provided in our GitHub repository.

For the surrogate models, we combine 40,500 FDTD simulations for micropyramids made of 41 different materials to form our training, validation, and test dataset. We follow a 70/20/10 percentage split respectively. The test dataset is used to evaluate the performance and overfitting of the model and it is not seen by the network in the training process. We shuffle the complete dataset every time the model is run or generated such that the training, validation, and test datasets are never identical from iteration to iteration.

For the inverse model, the training data is generated using the surrogate data. Whereas the surrogate provides the reflectivity and transmissivity provide an output for an individual wavelength point, the inverse uses a full vector input by stacking predictions from the surrogate. The full wavelength vector input that corresponds to an output we refer to as a “surrogate simulation”. For each material, we develop a grid of surrogate simulations by varying the xspan and zspan of the micropyramid and attaching a randomized wavelength vector and thickness value for each individual set of (xspan, zspan) in the grid. The grid is generated using a randomization process for pairs of x and z geometric coordinates. The process checks to ensure each material has no repeated pairs. The randomization process for the wavelength vector involves creating a linearly spaced vector of 100 points with a randomized minimum and maximum value. The minimum and maximum values are the randomly generated parameters and are between (0.3–15) and (2–16) μm respectively. If the randomly selected “minimum value” is larger than the “maximum” value, the values are switched in generating the wavelength vector. The random process is iterated to ensure that the gap between the minimum and maximum wavelength values is 2 um. The material information is generated from inputting the generated wavelength vector into a splined curve fit. The splined curve-fits are generated using a 2000-point dataset for each material. Due to the size of the inverse dataset, we adopt a 50/40/10 training/validation/test split for the inverse network training process.

### Post-processing: material fitting

The output of the inverse neural network contains a vector of n and k values, matched to an input of wavelength points. To provide the “best-fit” material, we compare the material data (n, k) to the material data in the library. The library data is generated using the same spline process as described in the previous section and depends on the user wavelength spectrum that was input into the reverse network. We check each (n, k) vector combination in the material library against the model output using the least-squares method shown in Eq. (5).

Before comparing the values using Eq. (5), we adjust the (n,k) vector using a log transformation shown in Eq. (6). While a comparison using Eq. (5) is still viable, the log adjustment allows for better comparisons to materials that depend strongly on minute differences in n, k values. As discussed, transmissive materials depend strongly on small changes in the n and k values, so having a scale that enables better comparison for small values enables us to draw better conclusions from Eq. 6as to which materials best match the ML output.

5 $$LSE = \mathop \sum \limits_{i = 2}^{n} \left( {Y_{i} - \widehat{{Y_{i} }}} \right)^{2}$$

6 $$\left( {n,k} \right)_{adj} = \log_{10} (\left( {n,k} \right) + a)$$

### Post-processing: new solution generation

In the post-processing module, we adjust the inverse network output according to user set constraints. In our present work, we only limit the aspect ratio, but the method can easily be adjusted to account for more conditions. To do this, we generate two sets of new geometric coordinates (for the same material) that do not violate the constraint. The process starts by adjusting the original solution’s x and z coordinate while fixing the other coordinate until the aspect ratio is within the constraint’s bounds. This follows our previously established intuition on the role of aspect ratio in determining optical/thermal property optimality^20^. For a different microstructure, we would need to adjust this process to match the observed patterns for the microtexture and the desired constraint. From these two new points, we generate new geometric pairs within a radius around the modified geometric coordinate. The generation process is random and uniform, and solutions that are not below or equal to the desired aspect ratio are eliminated. All viable solutions are then passed into the surrogate for predictions; the results that best match the desired input are simulated in FDTD. We also select random points from the remaining pool of randomly generated viable geometric solutions to have additional solution variety and to reduce concerns of over-biasing the network when the surrogate incorporates the new FDTD solutions in training. The desired input and model/FDTD outputs are evaluated for optimality using Eq. (5). This process is used to generate solutions separately for the direct ML material output and then the “best-fit” material(s). We will often only use a single “best-fit” material, but for some cases we will look beyond the first library match.

## Supplementary Information

Supplementary Information.
