# Supplementary material for: Deep learning-based inverse design of microstructured materials for optical optimization and thermal radiation control
Source: Sci Rep. 2023 May 6;13:7382. doi: 10.1038/s41598-023-34332-3 (PMC10164128; doi:10.1038/s41598-023-34332-3)
Supplement: Supplementary file 1 — Supplementary Information. [file 41598_2023_34332_MOESM1_ESM.docx]

**Deep Learning-based Inverse Design of Microstructured Materials for Optical Optimization and Thermal Radiation**

Authors: Jonathan Sullivan^a^, Arman Mirhashemi^b^, Jaeho Lee^a*^

*^a^Department of Mechanical and Aerospace Engineering, University of California Irvine, United States*

*^b^NASA Glenn Research Center, Cleveland OH, United States*

*Corresponding author: jaeholee@uci.edu

**Supplementary Information**

**Section S1. Material Data and Material Library**

A wide array of materials are utilized in both the training of the neural network and the prediction of optical properties. How the model differentiates between materials is the same as an optical simulation, by discerning different values of the complex refractive index. Whereas in our previous work, we selected a variety of materials for the training, ranging from metals (Ni/Ag/Al/Cr/Fe/Sn), refractory metals (Ta/W), a phase-change material (VO2 Metallic/Insulating), a polymer (PDMS), and a semiconductor (SiC), we utilize all available simulations to train the surrogate in this work. In total, we utilize a library of 50 materials for training the neural network and making grid predictions. The comprehensive list of materials and refractive index references are shown in Table S1. The size of each material’s simulation dataset is noted in Table S1. The largest group of materials (previously “unseen” materials for our surrogate network^1^) have 100 simulations each, which are enough to calibrate the model to an accuracy near to the accuracy demonstrated for materials with much larger datasets included in training. It should be noted that these materials were not chosen based on experimental validity or practicality of manufacture. Materials are chosen on a basis of difference from other materials and availability of material data over a wide spectrum of wavelengths. Not all material information extends from 0.3 to 16 um (the wavelength min/max used in training), so we extrapolate material information to cover the wavelengths that were not incorporated in the original material information. Material limitations can be imposed, however, in the material search process as part of the aggregate inverse network’s post-processing functionality. Ad

| **Original Surrogate Materials*** | **Library Materials**** |
| --- | --- |
| Alumina (Al2O3)^2^ | Aluminum Nitride (AlN)^2^ |
| Aluminum (Al)^3^ | Boron Carbide (B4C)^4^ |
| Chromium (Cr)^5^ | Barium Fluoride (BaF2)^6^ |
| Copper (Cu)^5^ | Beryllium (Be)^7^ |
| Diamond^8^ | Carbon (Graphite)^9^ |
| Iron (Fe)^5^ | Carbon (Graphene)^10^ |
| Nickel (Ni)^3^ | Copper (Cu)^5^ |
| PDMS^11^ | Calcium Magnesium Carbonate (CaMg(CO3)2) |
| Silver (Ag)^12^ | Cesium Bromide (CsBr) |
| Silicon Carbide (SiC)^13^ | Gallium Arsenide (GaAs)^5^ |
| Silicon Dioxide (SiO2)^5^ | Germanium (Ge) |
| Tin (Sn)^5^ | Gold (Au)^14^ |
| Tantalum (Ta)^15^ | Indium (In)^16^ |
| Titanium (Ti)^5^ | Indium Arsenide (InAs)^17^ |
| Vanadium Dioxide (M)^18^ | Indium Phosphate (InP)^17^ |
| Vanadium Dioxide (I)^18^ | Lithium (Li)^19^ |
| Tungsten (W)^5^ | Lead (Pb)^6^ |
|  | Magnesium (Mg)^20^ |
|  | Molybdenum (Mo)^15^ |
|  | Niobium (Nb)^21^ |
|  | Osmium (Os)^22^ |
|  | Potassium Chloride (KCl) |
|  | Palladium (Pd)^5^ |
|  | Platinum (Pt)^5^ |
|  | Rhodium (Rh)^5^ |
|  | Silicon (Si)^5^ |
|  | Silicon Nitride (Si3N4)^2^ |
|  | Titanium Dioxide (TiO2)^23^ |
|  | Vanadium (V)^3^ |
|  | Zinc (Zn)^6^ |
|  | Zinc Oxide (ZnO)^6^ |
|  | Zinc Sulfide (ZnS)^6^ |
|  | Zinc Selenide (ZnSe) ^6^ |
|  | Zirconium (Zr)^6^ |

* Each material in this category has at least 1000 simulations

* Each material in this category has exactly 100 simulations included as baseline

**Section S2. Inverse Predictions with Modified Transmission Input**

While we constrain the transmission to a value of 0 in the case of the “ideal” emitters, as discussed in the main body, this assumption can be unrealistic, and the model is forced to find comparable solutions – this process yields a result that is a compromise between the transmission and the desired emissivity. When we consider a single material case, however, for an ideal cooling material where T = 1 – E and R = 0, we receive the anticipated material output, as shown in Figure S1. The matched material outputs are PDMS and SiO2, well established thermal emitters, and the texturing provides optimal emission characteristics in the infrared. This comes at the cost of transmission in the NIR because of the material properties. When we enable the model to search with a modified transmission value, we receive the anticipated result for a material that closely follows the desired emission behavior.


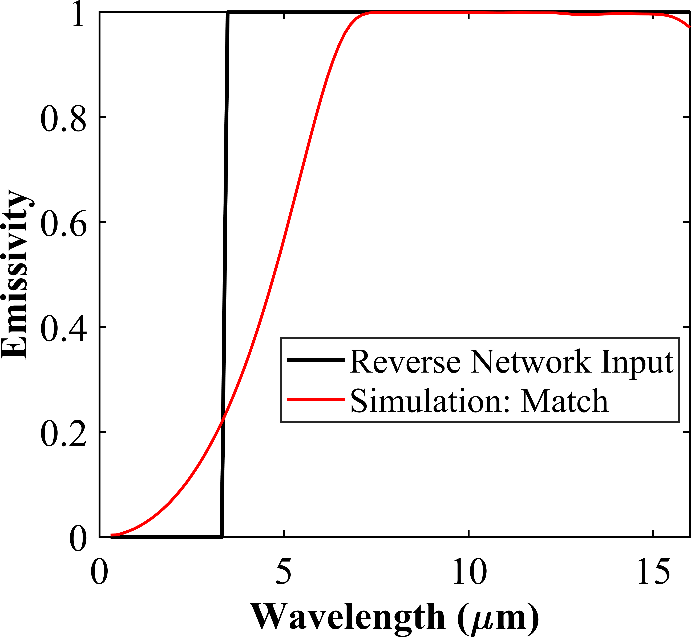


**Figure S1** FDTD Simulation for optimized PDMS geometry, when the input transmission for the ideal cooling case is set to be T = 1 – E, as opposed to 0


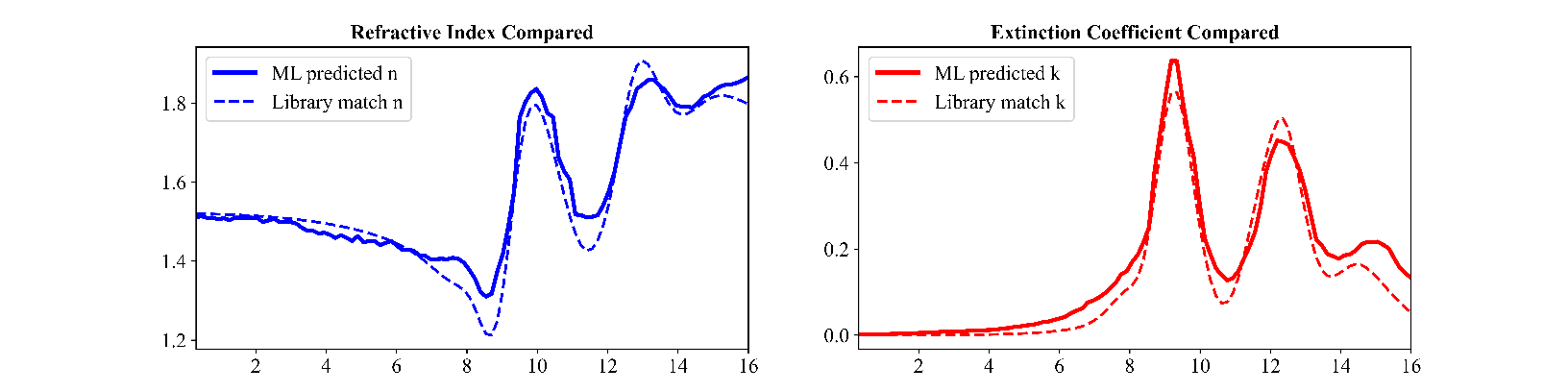


**Figure S2** (a) Refractive Index and (b) Extinction coefficient output by the inverse neural network. The material outputs nearly identically match those of PDMS

The optimal geometry identified for this input spectrum is PDMS with a base dimension of 0.52 um and a height of 7.34 um. When simulated, the results plotted in Figure S2 closely match expectation and input, with an emission that is near to 100% in the infrared.

**Section S3. Textured Comparisons to Flat Materials**

Texturing can be more numerically or superficially impactful for materials where a microstructure significantly enhances an optical response. This is visible in the optimized metal case shown in Figure S3, the difference between textured Li and flat Li is instantly recognizable. Untextured Li has an average absorption of ~ 0.14 in the solar region, whereas textured Li has an average absorption of ~ 0.96, representing a significant increase in terms of absorbed solar radiation. Comparatively, silicon nitride is already an emissive material, so while texturing does increase the emission value, the relative difference is less extreme than the ideal heating case. It should be noted for the textured graphite case that when we simulate “flat” graphite we are simulating the same thickness for the substrate and assuming that the surface is perfectly flat. In practice, graphite is highly emissive as the surface exhibits high degrees of surface roughness and is often much thicker than the 0.1 mm (100 um) maximum thickness output of the neural network. The computed absorption/emission values are computed relative to the desired region of influence. That is, for the ideal heating, alpha is computed as the amount of absorbed power relative to the maximum solar radiation. For ideal cooling and unity emissivity case, the emission is computed as the thermal emission vs the maximum thermal emission.. The precise values for all of the categories are shown below in Table S2.


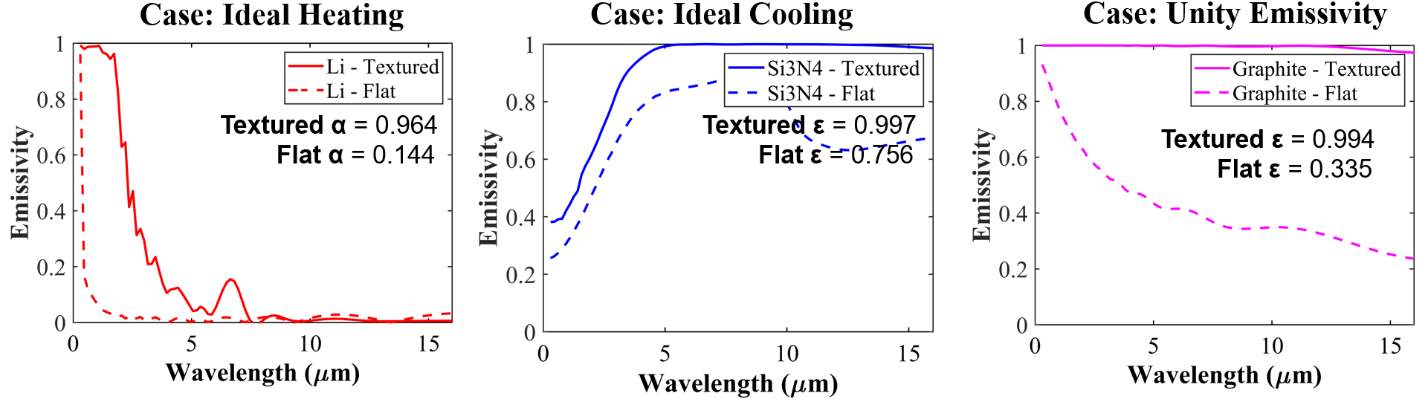


**Figure S3** Comparing the optical properties of untextured materials to the inverse network optimized textured materials

|  |  |  |  |  |  |
| --- | --- | --- | --- | --- | --- |
|  | Absorption  Efficiency | Emission  Efficiency | Power  Absorbed | Power  Emitted | Emissivity Deviation  from Input (MSE) |
| **Flat** |  |  |  |  |  |
| Graphite | 0.8176 | 0.3356 | 817 | 93.57 | 0.3658 |
| Li | 0.1438 | 0.0161 | 144 | 4.5 | 0.1256 |
| Si3N4 | 0.3069 | 0.7557 | 307 | 210.7 | 0.092 |
|  |  |  |  |  |  |
| **Textured** |  |  |  |  |  |
| Graphite | 0.9999 | 0.9943 | 1000 | 277.3 | 6.62E-05 |
| Li | 0.964 | 0.0225 | 964 | 6.26 | 0.0155 |
| Si3N4 | 0.425 | 0.9969 | 425 | 277.98 | 0.0656 |

**Table S2** Comparing untextured materials to the optimized texturing demonstrated in the main text. Texturing has a significant impact in some cases, but the transformation is most apparent in the case of the reflective metal turned into a solar absorbing material (Li).

**References**

1. Sullivan, J., Mirhashemi, A. & Lee, J. Deep learning based analysis of microstructured materials for thermal radiation control. *Sci. Rep.* **12**, 1–14 (2022).

2. Kischkat, J. *et al.* Mid-infrared optical properties of thin films of aluminum oxide, titanium dioxide, silicon dioxide, aluminum nitride, and silicon nitride. *Appl. Opt.* **51**, 6789–6798 (2012).

3. Weber, M. J. *Handbook of Optical Materials*. *CRC Press* vol. 3 (2003).

4. Larruquert, J. I. *et al.* Self-consistent optical constants of sputter-deposited B 4 C thin films. **29**, 117–123 (2012).

5. Palik, E. D. *Handbook of Optical Constants of Solids*. (Academic Press, 1985).

6. Querry, M. R. From the Millimeter To the Ultraviolet. (1987).

7. Rakić, A. D., Djurišić, A. B., Elazar, J. M. & Majewski, M. L. Optical properties of metallic films for vertical-cavity optoelectronic devices. *Appl. Opt.* **37**, 5271 (1998).

8. Phillip, H. R. & Taft, E. A. Kramers-Kronig Analysis of Reflectance Data for Diamond. *Phys. Rev.* **136**, (1964).

9. Querry, M. R. Optical Constants, Report No. AD-A158 623. *Crdc* **CR**-**85034**, 1–413 (1985).

10. Falkovsky, L. A. Optical properties of graphene. *J. Phys. Conf. Ser.* **129**, 2–6 (2008).

11. Srinivasan, A., Czapla, B., Mayo, J. & Narayanaswamy, A. Infrared dielectric function of polydimethylsiloxane and selective emission behavior. *Appl. Phys. Lett.* **109**, (2016).

12. Yang, H. U. *et al.* Optical dielectric function of silver. *Phys. Rev. B - Condens. Matter Mater. Phys.* **91**, 1–11 (2015).

13. Larruquert, J. I. *et al.* Self-consistent optical constants of SiC thin films. *J. Opt. Soc. Am. A* **28**, 2340 (2011).

14. Babar, S. & Weaver, J. H. Optical constants of Cu, Ag, and Au revisited. *Appl. Opt.* **54**, 477 (2015).

15. Ordal, M. A., Bell, R. J., Alexander, R. W., Newquist, L. A. & Querry, M. R. Optical properties of Al, Fe, Ti, Ta, W, and Mo at submillimeter wavelengths. *Appl. Opt.* **27**, 1203 (1988).

16. Koyama, R. Y., Smith, N. V. & Spicer, W. E. Optical properties of indium. *Phys. Rev. B* **8**, 2426–2432 (1973).

17. You, A., Be, M. A. Y. & In, I. Optical dispersion relations for GaP, GaAs, GaSb, InP, InAs, InSb, Al. **6030**, (1998).

18. Wan, C. *et al.* On the Optical Properties of Thin‐Film Vanadium Dioxide from the Visible to the Far Infrared. *Ann. Phys.* **1900188**, 1900188 (2019).

19. Rasigni, M. & Rasigni, G. Optical constants of lithium deposits as determined from the Kramers-Kronig analysis. *J. Opt. Soc. Am.* **67**, 54 (1977).

20. Hagemann, H. J., Gudat, W. & Kunz, C. OPTICAL CONSTANTS FROM THE FAR INFRARED TO THE X-RAY REGION: Mg, Al, Cu, Ag, Au, Bi, C, and Al2O3. *J Opt Soc Am* **65**, 742–744 (1975).

21. Golovashkin, A. I., Leksina, I. E., Motulevhich, G. P. & Shubin, A. A. The optical properties of Niobium. *Sov. Phys. JetP* **29**, 27–34 (1969).

22. Nemoshkalenko, V. V, Antonov, V. N., Kirillova, M. M., Krasovskii, A. E. & Nomerovannaya, L. V. The Structure and Energy Bands and Optical Absoprtion in Osmium. *Sov. JetP* **63**, 115 (1986).

23. Siefke, T. *et al.* Materials Pushing the Application Limits of Wire Grid Polarizers further into the Deep Ultraviolet Spectral Range. *Adv. Opt. Mater.* **4**, 1780–1786 (2016).
